# Testosterone therapy and cardiovascular events among men: a systematic review and meta-analysis of placebo-controlled randomized trials

**DOI:** 10.1186/1741-7015-11-108

**Published:** 2013-04-18

**Authors:** Lin Xu, Guy Freeman, Benjamin J Cowling, C Mary Schooling

**Affiliations:** 1 School of Public Health, Li Ka Shing Faculty of Medicine, The University of Hong Kong, 21 Sassoon Road, Pokfulam, Hong Kong, China; 2 CUNY School of Public Health at Hunter College, 2180 Third Avenue, New York, NY, 10035, USA

**Keywords:** Testosterone, Cardiovascular, Men, Trial

## Abstract

**Background:**

Testosterone therapy is increasingly promoted. No randomized placebo-controlled trial has been implemented to assess the effect of testosterone therapy on cardiovascular events, although very high levels of androgens are thought to promote cardiovascular disease.

**Methods:**

A systematic review and meta-analysis was conducted of placebo-controlled randomized trials of testosterone therapy among men lasting 12+ weeks reporting cardiovascular-related events. We searched PubMed through the end of 2012 using “(“testosterone” or “androgen”) and trial and (“random*”)” with the selection limited to studies of men in English, supplemented by a bibliographic search of the World Health Organization trial registry. Two reviewers independently searched, selected and assessed study quality with differences resolved by consensus. Two statisticians independently abstracted and analyzed data, using random or fixed effects models, as appropriate, with inverse variance weighting.

**Results:**

Of 1,882 studies identified 27 trials were eligible including 2,994, mainly older, men who experienced 180 cardiovascular-related events. Testosterone therapy increased the risk of a cardiovascular-related event (odds ratio (OR) 1.54, 95% confidence interval (CI) 1.09 to 2.18). The effect of testosterone therapy varied with source of funding (*P*-value for interaction 0.03), but not with baseline testosterone level (*P*-value for interaction 0.70). In trials not funded by the pharmaceutical industry the risk of a cardiovascular-related event on testosterone therapy was greater (OR 2.06, 95% CI 1.34 to 3.17) than in pharmaceutical industry funded trials (OR 0.89, 95% CI 0.50 to 1.60).

**Conclusions:**

The effects of testosterone on cardiovascular-related events varied with source of funding. Nevertheless, overall and particularly in trials not funded by the pharmaceutical industry, exogenous testosterone increased the risk of cardiovascular-related events, with corresponding implications for the use of testosterone therapy.

## Background

In observational studies low serum testosterone is associated with cardiovascular disease [1,2]. Testosterone may protect or be a secondary risk marker of other processes [1-3]. On the precautionary principle, expert advice and reviews, largely based on observational evidence, warn that cardiovascular disease may be increased by androgen deprivation therapy [4] and low testosterone [5,6]. Awareness of low testosterone as a treatable condition is being raised [7,8]. Testosterone use is increasing [9-11], possibly as self-medication in response to advertising.

In 2004 the Institute of Medicine (IOM) reviewed the evidence on testosterone therapy and concluded, largely based on placebo-controlled trials, that ‘there is not clear evidence of benefit for any of the health outcomes examined’ [12]. The IOM recommended small-scale trials to establish the efficacy of testosterone therapy where other treatments were not available [12]. To our knowledge, no trial has been designed to assess the effect of testosterone therapy on cardiovascular morbidity or mortality. Previous meta-analyses of randomized placebo-controlled trials found that testosterone therapy resulted in a non-significantly higher risk of cardiovascular events, based on adverse events, but only included trials through March 2005 [13,14]. A more recent meta-analysis included trials through August 2008 but only reported on three specific cardiovascular outcomes, that is, arrhythmia, coronary bypass surgery and myocardial infarction [15]. Given the widespread use of testosterone, the high prevalence of cardiovascular disease in older men and no comprehensive assessment of the effect of testosterone therapy on cardiovascular events, an up-to-date meta-analysis may help inform clinical practice. We carried out a meta-analysis of adverse events from randomized placebo-controlled trials to examine the overall risk of cardiovascular-related events associated with testosterone therapy.

## Methods

This meta-analysis follows the Preferred Reporting Items for Systematic Reviews and Meta-Analyses (PRISMA) checklist [see Additional file 1] and a published protocol (CRD42011001815) [16]. Two reviewers (LX and CMS) independently searched for and selected trials, resolving any differences by consensus. Two statisticians (GF and BJC) extracted information from the selected trials.

### Data sources and searches

We (LX and CMS) systematically searched PubMed until 31st December 2012 using “(“testosterone” or “androgen”) and (random*) and trial” with the selection limited to studies in men in English, because a preliminary search only found studies in English. We (LX and CMS) searched the World Health Organization (WHO) International Clinical Trials Registry Platform for any trial using testosterone as an intervention. From this search, we (LX and CMS) discarded any studies both agreed were irrelevant based on title or abstract and read the remaining. We did a bibliographic search of the selected trials and relevant reviews.

### Study selection

We included randomized placebo-controlled trials giving cardiovascular-related events by study arm, because a report may focus on a particular aspect of the trial [17,18] and not report all events that have occurred [17-19]. We excluded trials that only gave treatment-related events in the testosterone arm because these might not include full reporting of events in the placebo arm. Initially, we intended to exclude trials that only reported withdrawals as potentially less comprehensive than reporting of adverse events [18,19], however this turned out to be a very fine distinction, so we included any trial reporting cardiovascular-related events by study arm.

We included any randomized controlled trial (RCT) of testosterone, but not other androgens, compared with placebo, including a comparison against a background of other treatments, because men likely to be taking testosterone may also be in treatment for other conditions. We excluded trials of less than twelve weeks’ duration to assess long-term rather than acute effects of testosterone therapy.

We checked for duplication based on overlapping authorship, study description, number of participants and participant characteristics. When duplication occurred we used the study with the most comprehensive description of adverse events.

### Outcome

The primary outcome was composite cardiovascular-related events; because we anticipated too few events for robust assessment by cardiovascular event type; and a system-wide composite outcome may be most suitable for adverse events [20]. Cardiovascular-related events were defined as anything reported as such by the authors, that is, events reported as cardiac disorders, cardiovascular complaints, cardiovascular events, vascular disorders, cardiac or cardiovascular, or where the event description fell within the International Statistical Classification of Disease (ICD) version 10 chapter IX (I00 to I99). Most trials only reported serious adverse events, but a few also reported a wider range of events, so we also examined the effect of testosterone therapy by seriousness. Seriousness was based on the US Department of Agriculture definition of serious adverse events and the type of cardiovascular event generally considered serious [21]. Serious cardiovascular events were defined as cardiovascular-related events which the authors described as serious adverse events or where the outcome was death, life-threatening, hospitalization, involved permanent damage or required medical/surgical intervention, or was one of the following types of cardiovascular event: myocardial infarction, unstable angina, coronary revascularization, coronary artery disease, arrhythmias, transient ischemic attacks, stroke or congestive heart failure but not deep vein thrombosis.

### Data extraction and quality assessment

A statistician (GF) extracted the number of participants randomized and cardiovascular-related events by trial arm. Event classification was checked by a physician (LX). A second statistician (BJC) checked the information extracted. Where trials reported cardiovascular-related events without giving the study arm, we contacted the authors twice by email to ask for cardiovascular-related events by study arm.

The reviewers (LX and CMS) independently used an established tool to evaluate the quality of each trial [22], focusing on the quality of reporting of cardiovascular-related adverse events. First, we reported whether cardiovascular-related events were individually listed in a table by study arm, because these are easier to identify unambiguously. Second, we reported whether the type and severity of cardiovascular-related events reported was either pre-specified or identified before the allocation was revealed, because issues have been expressed about the reporting of adverse events [23,24]. Cardiovascular-related events vary in severity making the selection criteria and categorization crucial to an outcome assessed from adverse events.

### Sensitivity analysis

We initially planned only to assess whether the effects of testosterone on cardiovascular-related events varied with average baseline testosterone, because we did not expect sufficient trials for sub-group analysis by type of testosterone product or by type of cardiovascular-related event. However, the reporting of adverse events may be open to interpretation [23], and may not be comprehensive [25]. Given potential lack of clarity as to the selection of the cardiovascular-related adverse events reported, we also examined whether the effect of testosterone therapy varied with funding source. Finally, we also considered cardiovascular-related death as an outcome.

### Data synthesis and analysis

We used the number of participants randomized as the denominator and included all cardiovascular-related events from the start. We used funnel plots and ‘trim and fill’ to assess publication bias, that is, missing trials. We used I^2^ to assess heterogeneity between trials, using fixed effects models where there was low heterogeneity (I^2^ <30%), otherwise using random effects models. We obtained the pooled odds ratio, using the ‘metabin’ function of the ‘meta’ package in R 2.14.1 (R Development Core Team, Vienna, Austria). We used meta-analysis regression, with inverse variance weighting, to assess whether the effects of testosterone therapy varied with baseline testosterone or funding source, using the ‘rma’ function of the ‘metafor’ package in R 2.14.1. Initial analysis showed the pooled odds ratio was similar using a Peto or a Mantel-Haenszel estimate; we used inverse variance weighting for consistency with the meta-regression.

This study is an analysis of published data, which does not require ethics committee approval.

## Results

**Figure 1:**
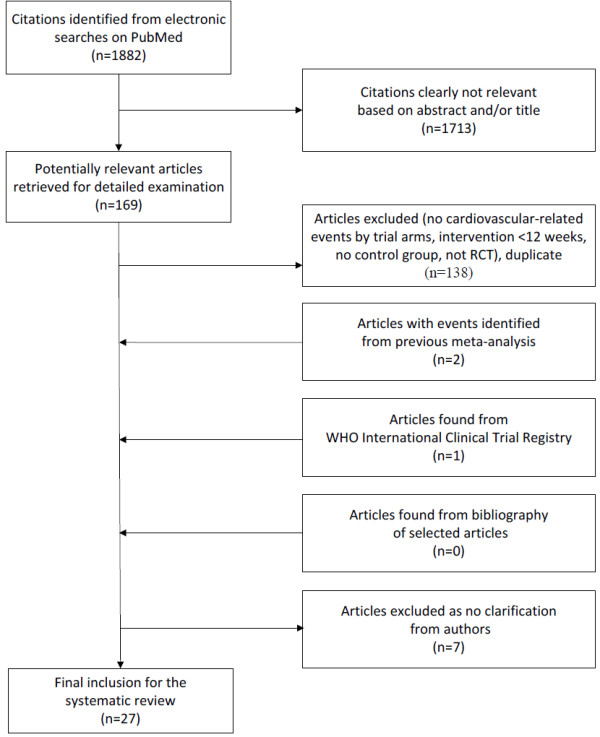
Selection process for the placebo-controlled randomized trials of the effects of testosterone therapy on cardiovascular-related events.

The initial search yielded 1,882 papers, of which 169 were selected for full text scrutiny. Of these 169 papers, 31 concerned different placebo-controlled randomized trials among men of testosterone therapy of 12+ weeks reporting cardiovascular-related events by study arm. We found one additional recent trial from the WHO International Clinical Trials Registry Platform [26]. We did not find any additional such trials from a bibliographic search of these 32 papers or from eight reviews [27-34]. Two additional trials [35,36] selected for full text scrutiny had cardiovascular-related events shown in a plot from one previous meta-analysis [13], but not the other meta-analyses [14,15] or in the relevant publications [35-37]. The search did not find one small non-randomized trial [38] included in two previous meta-analyses [13,15]. The search found one additional trial [39] potentially relevant to the earlier meta-analyses [13,14], two additional trials [40,41] potentially relevant to the most recent meta-analysis [15] and 11 subsequent trials. We sought clarification concerning events by study arm for 10 trials as set out in Additional file 2. Six authors never responded [37,40,42-45], three responded but did not provide any relevant information [36,46,47] and one provided information [48]. We included this later trial [48], one of the others that gave cardiovascular deaths, but not other cardiovascular-related events, by study arm [46] and one that gave vascular events, but not all cardiovascular-related events by study arm [44]. Figure 1 shows the search strategy resulting in 27 placebo-controlled randomized trials.

Table 1 shows the 27 trials over 25 years of 2,994 mostly middle-aged or older men (1,733 testosterone and 1,261 placebo) with low testosterone and/or chronic diseases, who experienced 180 cardiovascular-related events. Most of the trials were in Western settings. Thirteen trials were supported by the pharmaceutical industry. Two trials were stopped early [49,50], one because of adverse events among the men allocated to testosterone [50] and one because ‘it would not be feasible to demonstrate - in the foreseeable future - a beneficial effect of testosterone [on mortality] by continuing the study’ [49].

**Table 1:** Characteristics of placebo-controlled randomized trials giving the effects of testosterone therapy on cardiovascular-related events among men

**Author and publication year**	**Study**				**Participants**				**Industry funding**
	**Setting**	**Duration**	**Dose**	**Cardiovascular-related events based on**	**Age range**	**Number**	**Health status**	**Initial T (nmol/L)**	
Copenhagen Study Group [49] 1986	Denmark	About 16 months^a^	200 mg/8 h micronized T, PO	Deaths	24 to 79	221	alcoholic cirrhosis	about 20	None given
Marin [39] 1993	Denmark	9 months	T gel 125 mg/day	Withdrawals	40 to 65	21	Obese, low T	15.3	Funded by Besins Iscovesco
Hall [51] 1996	UK	9 months	T enanthate 250 mg /month, IM	Withdrawals	34 to 79	35	Rheumatoid arthritis	16.1	Funded by Schering Healthcare
Sih [52] 1997	US	12 weeks	T cypionate IM every 14 to 17 days	Withdrawals	51 to 79	32	T<60 ng/dl	9.2	None given
English [53] 2000	UK	12 weeks	Transdermal T 5 mg/day	Withdrawals and safety data	Mean 62	50	Coronary artery disease	12.9	Patches given by Smith Kline Beecham
Snyder [19] 2001	US	36 months	Transdermal T 6 mg/day	Clinically apparent from hospital records	65+	108	Men with T one SD <475 ng/dl	12.7	Patches given by ALZA Corporation
Amory [54] 2004	US	36 months	T enanthate 200 mg/2 weeks, IM	Serious adverse cardiovascular events	65 to 83	48	TT <350 ng/dl	10	None given
Kenny [55] 2004	US	12 weeks	T enanthate 200 mg/3 weeks, IM	General description	73 to 87	11	Cognitive decline, bioavailable T <128 ng/dl	14.1	None
Svartberg [56] 2004	Norway	26 weeks	T enanthate 250 mg/month, IM	General description	Mean 66	29	COPD	21.1	None
Brockenbrough [57] 2006	US	6 months	Transdermal T gel 10 g/day	Side effects and adverse events	Mean 56	40	Dialysis and TT <300 ng/dl	7.3	Supported by Auxilium Pharmaceuticals
Malkin [58] 2006	UK	12 months	Transdermal T patch 5 mg/day	Serious adverse events	Mean 64	76	Heart failure	13.0	Medication given by Watson Pharmaceuticals
Merza [59] 2006	UK	6 months	Transdermal T patch 5 mg/day	Withdrawals	40+	39	TT <10 nmol/L	8.0	Supported by Ferring Pharmaceuticals Ltd
Nair [60] 2006	US	24 months	Transdermal T patch 5 mg/day	Adverse events	60+	62	DHEA<1.57 μg/ml, bioavailable T <103 ng/dl	13.7	Supported by The Endocrine Society
Emmelot-Vonk [61] 2008	Netherlands	6 months	TU 160 mg/day, PO	Adverse events	60 to 80	237	TT<13.7 nmol/L	10.7	Medication given by Organon NV
Svartberg [41] 2008	Norway	52 weeks	TU 1000 mg, MI at 0, 6, 16, 28 and 40 weeks	Deaths	60 to 80	38	TT≤11.0 nmol/L	8.3	Grant from Bayer Schering Pharma AG
Caminiti [62] 2009	Italy	12 weeks	TU 1000 mg MI for 0, 6 and 12 weeks	General description of events	66 to 76	70	Heart failure	7.0	None given
Chapman [48] 2009	Australia	1 year	TU 80 mg orally twice a day	Hospitalizations	65+	23	Undernourished	18.8	Organon provided funding
Legros [46] 2009	Europe	1 year	TU 80, 160 and 240 mg orally per day	Safety assessments	50+	316	Free T<0.26 nmol/L	12.8	Funded by Schering-Plough
Aversa [63] 2010	Italy	24 months	TU 1,000 mg (every 12 weeks)	Safety aspects	45 to 65	50	MS and/or T2DM TT<3.0 ng/ml	8.5	None given
Basaria [50] 2010	US	About 6 months^a^	Transdermal T gel 100 mg/day	Cardiovascular-related events	65+	209	Frial, TT 100 to 350 ng/dl	8.4	Medication given by Auxilium Pharmaceuticals
Srinivas-Shankar [64] 2010	US	6 months	Transdermal T gel 50 mg/day	Serious adverse events and withdrawals	65+	274	TT≤12 nmol/L (345 ng/dl)	11.0	Supported by Bayer Schering Pharma
Jones [65] 2011	Europe	12 months	T gel 60 mg/day	Cardiovascular events	37 to 88	220	Hypogonadal with type 2 diabetes and/or MetS,	9.4	Supported by ProStrakan
Ho 2011 [66]	Malaysia	1 year	TU 1000 mg MI for 0, 6, 18, 30 and 42 weeks	Withdrawals	40+	120	T<12 nmol/L,	9.0	Supported by Bayer Schering Pharma
Kaufman [44] 2011	US	182 days	1.62% T gel 2.5 mg/day	Safety aspects	45 to 64	274	Hypogonadal, T<300 ng/dl	9.8	Funded by Abbott.
Kalinchenko [67] 2010	Russia	30 weeks	TU 1,000 mg MI for 0, 6, 18 and 30 weeks	Withdrawal	35 to 70	184	T<350 ng/dl	7.0	Supported by Bayer Schering Pharma
Hoyos [68] 2012	Australia	18 weeks	TU 1000 mg MI at 0, 6 and 12 weeks	Adverse events	18+, mean 49	67	Obese men with obstructive sleep apnea	13.3	Supported by Bayer Schering Pharma
Spitzer [26] 2012	US	14 weeks	1% T gel 10 g/day	Adverse events	40 to 70	140	Erectile dysfunction low T and a sexual partner	12.3	none

^a^trial stopped early so duration varies. IM= intramuscularly; P, placebo; PO, orally; T= testosterone; TT= total testosterone; TU= testosterone undecanoate.

### Quality assessment

The quality assessment (see Additional file 3) shows that two trials provided a table with a comprehensive list of cardiovascular-related events by study arm and eight trials provided a summary table of cardiovascular-related events by study arm. For 17 trials cardiovascular-related events were surmised from withdrawals and/or adverse events as given in Additional file 4, including one where the cardiovascular-related events were surmised from a *P*-value [65]. The type and severity of adverse events to be reported was pre-specified in one trial [61]. In the two trials terminated early the adverse events motivated termination and were identified before treatment allocation was known [49,50]. Otherwise it was sometimes unclear whether the definition or classification was made by masked assessors.

### Data synthesis

**Figure 2:**
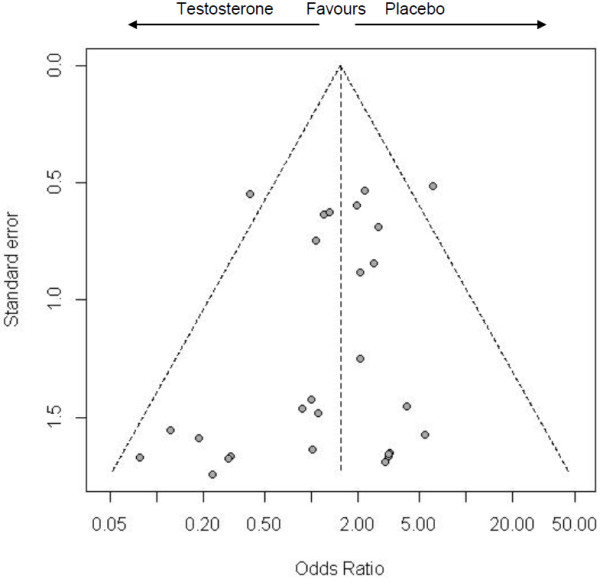
Funnel plot of placebo-controlled randomized trials examining the effects of testosterone therapy on cardiovascular-related events.

**Figure 3:**
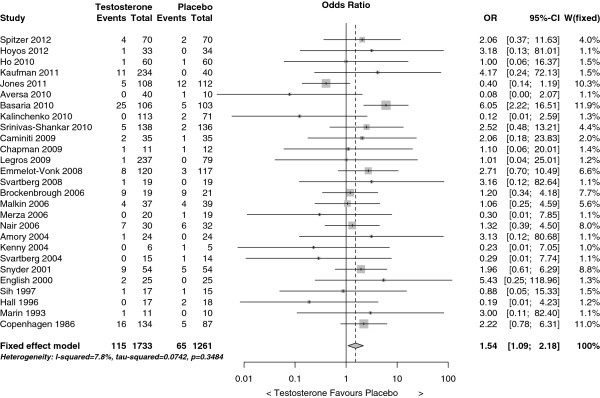
Forest plots of placebo-controlled randomized trials examining the pooled effect of testosterone therapy on cardiovascular-related events.

The funnel plot (Figure 2) shows several small studies (on the left hand side) where testosterone reduced cardiovascular-related events, however, there were no similar small studies where testosterone increased cardiovascular-related events. The forest plot (Figure 3) shows that the trials were homogeneous (I^2^ = 7.8%). Testosterone increased the risk of a cardiovascular-related event in a fixed effect model, odds ratio (OR) 1.54, 95% confidence interval (CI) 1.09 to 2.18. Trim and fill revised the OR to 1.69 (95% CI 1.21 to 2.38). When the analysis was restricted to serious events, whose categorization is shown in Additional file 4, the estimate was very similar (OR 1.61 (95% CI 1.01 to 2.56)) and was revised to 2.01 (95% CI 1.30 to 3.14) by trim and fill.

### Sensitivity analysis

**Figure 4:**
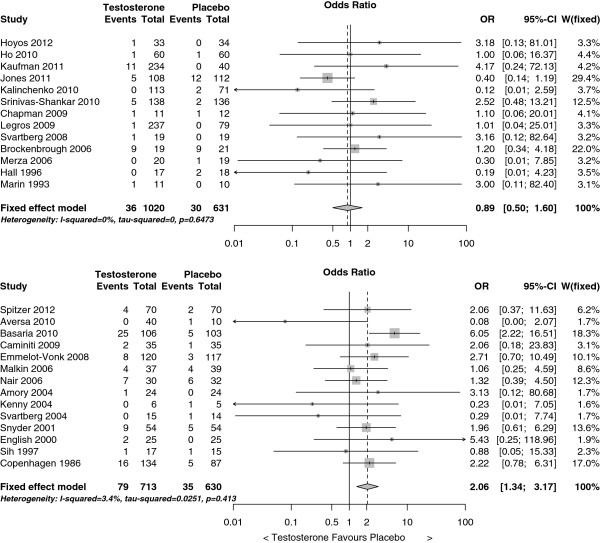
Forest plots of placebo-controlled randomized trials examining the pooled effects of testosterone therapy on cardiovascular-related events by source of funding: upper panel funded by the pharmaceutical industry and lower panel not funded by the pharmaceutical industry.

The cardiovascular-related event rate was lower in trials funded by the pharmaceutical industry (4% (66/1,651)) than in other trials (8% (114/1,343)). In a meta-regression model, risk of cardiovascular-related events on testosterone therapy varied with the source of funding (*P*-value for interaction 0.03) but not with baseline testosterone (*P*-value for interaction 0.70). In trials funded by the pharmaceutical industry testosterone had no effect on cardiovascular-related events, but in the other trials testosterone therapy substantially increased the risk of a cardiovascular-related event (Figure 4). Finally, 33 cardiovascular-related deaths were identified (22 testosterone arm and 11 placebo arm), for which the odds ratio was similar 1.42 (95% CI 0.70 to 2.89) to the estimate for all cardiovascular-related events, and was revised to 1.57 (95% CI 0.78 to 3.13) by trim and fill.

## Discussion

This updated meta-analysis of placebo-controlled randomized trials, with a much larger number of participants than previous meta-analyses [13-15], adds to the previous findings by showing that testosterone therapy increases cardiovascular-related events among men. The risk of testosterone therapy was particularly marked in trials not funded by the pharmaceutical industry. The risks of cardiovascular-related events were similar by baseline testosterone.

Several possible explanations exist for our findings. First, not all trials of testosterone therapy reported all cardiovascular-related events by study arm [36,46]. Trials favoring testosterone may be unpublished. However, the funnel plot (Figure 2) and ‘trim and fill’ suggested trials favoring the placebo may be missing. Second, endogenous and exogenous testosterone may have different effects, with endogenous testosterone being protective, consistent with the observational evidence [1,2] and with testosterone declining with age when cardiovascular disease increases with age. However, a recent Mendelian randomization study, using genetic variants as an instrumental variable for endogenous testosterone, did not corroborate protective effects of endogenous testosterone on cardiovascular disease risk factors [69]. Another possibility is that serum testosterone is not a good indicator of androgen activity [70], as has long been suggested [71] and recently substantiated by the effective use of anti-androgens in prostate cancer at castrate levels of serum testosterone [72,73]. Third, endogenous testosterone may be beneficial, but other metabolites of exogenous testosterone, raised by testosterone therapy, such as estrogens or dihydrotestosterone, could mediate cardiovascular-related events. Exogenous estrogens do not protect men against cardiovascular disease [74]. However, higher free testosterone rather than higher estradiol appeared to mediate the cardiovascular events in a recent trial of testosterone therapy [75]. Few trials have examined the effects of dihydrotestosterone administration and have usually focused on prostate rather than cardiovascular effects [76-78]. The interplay of testosterone and dihydrotestosterone is complex and challenging to disentangle in RCTs [79]. Nevertheless, exogenous testosterone lowers HDL-cholesterol [15] and raises hemoglobin, hematocrit and thromboxane [15,80], all of which might contribute to cardiovascular disease. Thromboxane promotes clotting and blood vessel constriction. Natural experiments suggest that lower lifetime endogenous androgens are associated with a relatively lower risk of death from ischemic heart disease, based on legally castrated men [81] and men with Klinefelter’s syndrome [82]. Similarly, a meta-analysis of RCTs of androgen deprivation therapy found a non-significantly lower risk of cardiovascular mortality among men allocated to androgen deprivation [83], despite bias to the null by the competing risk of death from prostate cancer.

Our findings are consistent with the three previous meta-analyses [13-15], which all indicated a non-significantly higher risk of testosterone therapy for a composite cardiovascular outcome of the events considered, despite discrepancies in some studies [13,14]. This meta-analysis based on many more trials (27), many more men (2,994) and correspondingly more events (180) produced a similar, but more precise estimate, with the confidence interval no longer including no effect. The difference between the estimates by funding source is consistent with other observations [84,85] and could be due to different reporting of adverse events in industry funded trials. Differences by funding source could also be due to differences between trials. Industry funded trials reported fewer cardiovascular-related events, which reduces power although it should not affect the direction of effect. Industry funded trials tended to be in younger men. It is possible, although unusual, for the effects of treatment to be ‘crossed’ by age [86].

From a clinical perspective the issue is ensuring that the benefits of testosterone therapy outweigh the potential risks. Almost a decade after the IOM’s report [12] the efficacy of testosterone therapy for health outcomes where treatments are not already available remains uncertain. Testosterone compared to placebo could be beneficial for glucose metabolism [65], depression [87,88], sexual dysfunction [26,89], bone density [90] and HIV wasting syndrome [91,92], although whether testosterone is better than established treatments for these conditions has not been clearly established. Cardiovascular disease is common in typical users of testosterone therapy, that is, older men. The 10-year risk of a cardiovascular event for US men aged 65 to 69 years is about 28% [93]. Assuming the increased risk of cardiovascular-related events seen here with testosterone therapy would give a number needed to harm of at most 90 per year of testosterone therapy. As such, further research might focus on obtaining evidence without interventions, for example by confirming that the observed negative associations of serum testosterone with specific cardiovascular diseases extends to other androgen biomarkers, such as androgen glucuronides [94], and to other study designs less open to biases, such as Mendelian randomization. Moreover, a gene in the steroidogenesis pathway (*CYP17A1)* is reliably associated with coronary artery disease [95]. Establishing if *CYP17A1* acts, if at all, by raising or lowering androgens would also bring clarity. Finally, consideration could be given to whether further trials should be of agents that raise or lower testosterone.

### Strengths and limitations

Despite providing a meta-analysis of all known placebo-controlled randomized trials, limitations exist. First, the reporting of adverse events may be open to conflicts of interest [24]. The funnel plot and analysis by funding source are consistent with that possibility. A very large market is at stake [9]. Second, in a trial of a therapy, such as testosterone, which may change how men feel or their sex drive, some accidental unmasking may have occurred. Few trials assessed or reported this possibility. Third, some men in the testosterone arm stopped treatment because of increased prostate specific antigen or polycythemia, which would bias towards the null. Fourth, RCTs are not always tagged as such and could be missed [96]. However, we searched broadly and found several potentially eligible trials that had not been included in previous meta-analyses. Fifth, our study cannot include on-going trials, such as the Testosterone Supplementation and Exercise in Elderly Men trial (NCT00112151) and The Testosterone Trial (NCT00799617). However, these trials are not designed to assess the effect of testosterone on cardiovascular events and will take time to complete. If new trials show testosterone therapy to be strongly protective against cardiovascular disease, it would be against the general run of evidence to date making interpretation uncertain because of heterogeneity [97]. Sixth, the abstractors were not blinded. Seventh, most trials only reported fairly serious cardiovascular-related events, but the severity varied between trials, although for RCTs the reporting of events should be comparable within trials, and the events reported are more or less severe symptoms of cardiovascular disease on the pathway to cardiovascular mortality. An analysis restricted to events which could be identified as serious gave a similar estimate, but was limited by relying on events the authors had chosen to describe in detail by study arm and was revised upwards by trim and fill. Arguably, the standard definitions of event seriousness, including hospitalization, do not apply to frail older men, because they may be particularly prone to hospitalization. On the other hand, frail older people may also be most affected by any decrement to their already poor health, so hospitalization may represent a particularly significant event. Notably, an estimate based solely on deaths also had a similar point estimate, although the confidence interval included no effect because of low power. Eighth, two larger trials were terminated early [49,50] which reduces power and could affect the estimates. However, the terminations took place towards the end of the planned trials and did not specifically concern cardiovascular-related events. Nevertheless, early terminations may have slightly increased the estimate and widened the confidence intervals. However, the interpretation would, most likely, have been similar. Ninth, although meta-analyses are a mainstay of evidence-based medicine they may be less reliable than large RCTs. Meta-analysis may overstate the benefits of treatment [98] however, they are less prone to overstate the harms [98]. Subsequent large RCTs rarely reverse the direction of effect from meta-analysis [99]. Tenth, given the lack of detailed cardiovascular-event reporting secondary analysis by type of cardiovascular event was not possible. Such sub-group analysis would undoubtedly be etiologically valuable. However, from a public health perspective the issue is identifying side-effects, where a composite outcome relating to a particular system (here the cardiovascular system) has been recommended [20]. Finally, the difference observed by source of funding could just be chance variation; however, testosterone therapy increased the risk of cardiovascular-related events overall.

## Conclusions

Appropriately prescribed testosterone is undoubtedly beneficial. However, caution needs to be exercised to ensure that the associated health benefits of testosterone therapy outweigh the potential increased risk of cardiovascular-related events, particularly in older men where cardiovascular disease is common.

## Abbreviations

CI: confidence interval; CYP17A1: cytochrome P450 17A1; IOM: Institute of Medicine; ICD: International Statistical Classification of Disease; OR: odds ratio; PRISMA: Preferred Reporting Items for Systematic Reviews and Meta-Analyses; RCT: randomized controlled trial; WHO: World Health Organization.

Systematic review reference number CRD42011001815.

## Competing interests

All authors declare: no support from any organization for the submitted work; BJC has received research funding from MedImmune Inc., and consults for Crucell MV; no other relationships or activities exist that could appear to have influenced the submitted work.

## Authors’ contributions

LX carried out the systematic search and drafted the manuscript. GF and BJC did the data extraction and analysis; they also reviewed the manuscript critically. CMS originated the idea, carried out the systematic search and helped draft the manuscript. All authors had full access to all the data in the study and take responsibility for the integrity of the data and the accuracy of the data analysis. LX is the guarantor. All authors read and approved the final manuscript.

## Pre-publication history

The pre-publication history for this paper can be accessed here:

http://www.biomedcentral.com/1741-7015/11/108/prepub

## Supplementary Material

Additional file 1 PRISMA 2009 Checklist. Click here for file

Additional file 2 **Trials where authors contacted for additional information and responses.**[13,35-37,40,42-48,100]. Click here for file

Additional file 3 **Quality assessment of the selected placebo-controlled RCTs of the effects of testosterone therapy on cardiovascular-related events (CRE) **[19]**,**[26]**,**[39]**,**[41]**,**[44]**,**[46]**,**[48]**-**[68]. Click here for file

Additional file 4 **Description of cardiovascular-related events in the selected placebo-controlled RCTs **[19]**,**[26]**,**[39]**,**[41]**,**[44]**,**[46]**,**[48]**-**[67]**,**[101]. Click here for file

## References

[B1] Araujo AB Dixon JM Suarez EA Murad MH Guey LT Wittert GA Clinical review: endogenous testosterone and mortality in men: a systematic review and meta-analysis J Clin Endocrinol Metab 2011 96 3007 3019 10.1210/jc.2011-1137 21816776 PMC3200249

[B2] Ruige JB Mahmoud AM De Bacquer D Kaufman JM Endogenous testosterone and cardiovascular disease in healthy men: a meta-analysis Heart 2011 97 870 875 10.1136/hrt.2010.210757 21177660

[B3] Sartorius G Spasevska S Idan A Turner L Forbes E Zamojska A Allan CA Ly LP Conway AJ McLachlan RI Handelsman DJ Serum testosterone, dihydrotestosterone and estradiol concentrations in older men self-reporting very good health: the healthy man study Clin Endocrinol (Oxf) 2012 77 755 763 10.1111/j.1365-2265.2012.04432.x 22563890

[B4] Levine GN D’Amico AV Berger P Clark PE Eckel RH Keating NL Milani RV Sagalowsky AI Smith MR Zakai N Androgen-deprivation therapy in prostate cancer and cardiovascular risk: a science advisory from the American Heart Association, American Cancer Society, and American Urological Association: endorsed by the American Society for Radiation Oncology CA Cancer J Clin 2010 60 194 201 10.3322/caac.20061 20124400 PMC3049943

[B5] Jones TH Testosterone deficiency: a risk factor for cardiovascular disease? Trends Endocrinol Metab 2010 21 496 503 10.1016/j.tem.2010.03.002 20381374

[B6] Traish AM Saad F Feeley RJ Guay A The dark side of testosterone deficiency: III Cardiovascular disease. J Androl 2009 30 477 494 10.2164/jandrol.108.007245 19342698

[B7] Eggertson L Brouhaha erupts over testosterone-testing advertising campaign CMAJ 2011 183 E1161 E1162 21969410 10.1503/cmaj.109-4000 PMC3216426

[B8] Morgentaler A Testosterone for Life 2008 New York: McGraw-Hill

[B9] Sexual dysfunction as the last bastion of urological drug commercialisation within the pharmaceutical industry BJU Int 2011 107 1845 1846 21592281 10.1111/j.1464-410X.2011.10348.x

[B10] Handelsmann DJ **Pharmacoepidemiology of testosterone prescribing in Australia**, 1992–2010 Med J Aust 2012 196 642 645 10.5694/mja11.11277 22676880

[B11] Gan EH Pattman S Pearce S Quinton R Many men are receiving unnecessary testosterone prescriptions BMJ 2012 345 e5469 10.1136/bmj.e5469 22915724

[B12] Committee on Assessing the Need for Clinical Trials of Testosterone Replacement Therapy TESTOSTERONE AND AGING Clinical Research Directions 2004 Washington: The National Academies Press 25009850

[B13] Calof OM Singh AB Lee ML Kenny AM Urban RJ Tenover JL Bhasin S Adverse events associated with testosterone replacement in middle-aged and older men: a meta-analysis of randomized, placebo-controlled trials J Gerontol A Biol Sci Med Sci 2005 60 1451 1457 10.1093/gerona/60.11.1451 16339333

[B14] Haddad RM Kennedy CC Caples SM Tracz MJ Bolona ER Sideras K Uraga MV Erwin PJ Montori VM Testosterone and cardiovascular risk in men: a systematic review and meta-analysis of randomized placebo-controlled trials Mayo Clin Proc 2007 82 29 39 17285783 10.4065/82.1.29

[B15] Fernandez-Balsells MM Murad MH Lane M Lampropulos JF Albuquerque F Mullan RJ Agrwal N Elamin MB Gallegos-Orozco JF Wang AT Erwin PJ Bhasin S Montori VM Clinical review 1: adverse effects of testosterone therapy in adult men: a systematic review and meta-analysis J Clin Endocrinol Metab 2010 95 2560 2575 10.1210/jc.2009-2575 20525906

[B16] Schooling C Xu L Freeman G Cowling B Testosterone and cardiovasclar related events in men: a meta-analysis of randomized controlled trials PROSPERO 2011:CRD42011001815 http://www.crd.york.ac.uk/PROSPERO/display_record.asp?ID=CRD42011001815

[B17] Snyder PJ Peachey H Hannoush P Berlin JA Loh L Lenrow DA Holmes JH Dlewati A Santanna J Rosen CJ Strom BL Effect of testosterone treatment on body composition and muscle strength in men over 65 years of age J Clin Endocrinol Metab 1999 84 2647 2653 10.1210/jc.84.8.2647 10443654

[B18] Snyder PJ Peachey H Hannoush P Berlin JA Loh L Holmes JH Dlewati A Staley J Santanna J Kapoor SC Attie MF Haddad JG Jr Strom BL Effect of testosterone treatment on bone mineral density in men over 65 years of age J Clin Endocrinol Metab 1999 84 1966 1972 10.1210/jc.84.6.1966 10372695

[B19] Snyder PJ Peachey H Berlin JA Rader D Usher D Loh L Hannoush P Dlewati A Holmes JH Santanna J Strom BL Effect of transdermal testosterone treatment on serum lipid and apolipoprotein levels in men more than 65 years of age Am J Med 2001 111 255 260 10.1016/S0002-9343(01)00813-0 11566454

[B20] Tugwell P Judd MG Fries JF Singh G Wells GA Powering our way to the elusive side effect: a composite outcome ‘basket’ of predefined designated endpoints in each organ system should be included in all controlled trials J Clin Epidemiol 2005 58 785 790 10.1016/j.jclinepi.2004.11.028 16018913

[B21] Singh S Loke YK Spangler JG Furberg CD Risk of serious adverse cardiovascular events associated with varenicline: a systematic review and meta-analysis CMAJ 2011 183 1359 1366 21727225 10.1503/cmaj.110218 PMC3168618

[B22] Verhagen AP de Vet HC de Bie RA Kessels AG Boers M Bouter LM Knipschild PG The Delphi list: a criteria list for quality assessment of randomized clinical trials for conducting systematic reviews developed by Delphi consensus J Clin Epidemiol 1998 51 1235 1241 10.1016/S0895-4356(98)00131-0 10086815

[B23] Doshi P Jones M Jefferson T Rethinking credible evidence synthesis BMJ 2012 344 d7898 10.1136/bmj.d7898.:d7898 22252039

[B24] Ioannidis JP Adverse events in randomized trials: neglected, restricted, distorted, and silenced Arch Intern Med 2009 169 1737 1739 19858427 10.1001/archinternmed.2009.313

[B25] Pitrou I Boutron I Ahmad N Ravaud P Reporting of safety results in published reports of randomized controlled trials Arch Intern Med 2009 169 1756 1761 19858432 10.1001/archinternmed.2009.306

[B26] Spitzer M Basaria S Travison TG Davda MN Paley A Cohen B Mazer NA Knapp PE Hanka S Lakshman KM Ulloor J Zhang A Orwoll K Eder R Collins L Mohammed N Rosen RC DeRogatis L Bhasin S Effect of testosterone replacement on response to sildenafil citrate in men with erectile dysfunction: a parallel, randomized trial Ann Intern Med 2012 157 681 691 10.7326/0003-4819-157-10-201211200-00004 23165659

[B27] Corona G Rastrelli G Monami M Guay A Buvat J Sforza A Forti G Mannucci E Maggi M Hypogonadism as a risk factor for cardiovascular mortality in men: a meta-analytic study Eur J Endocrinol 2011 165 687 701 10.1530/EJE-11-0447 21852391

[B28] Cattabiani C Basaria S Ceda GP Luci M Vignali A Lauretani F Valenti G Volpi R Maggio M Relationship between testosterone deficiency and cardiovascular risk and mortality in adult men J Endocrinol Invest 2012 35 104 120 22082684 10.3275/8061

[B29] Carson CC 3rd Rosano G Exogenous testosterone, cardiovascular events, and cardiovascular risk factors in elderly men: a review of trial data J Sex Med 2012 9 54 67 10.1111/j.1743-6109.2011.02337.x 21676183

[B30] Gullett NP Hebbar G Ziegler TR Update on clinical trials of growth factors and anabolic steroids in cachexia and wasting Am J Clin Nutr 2010 91 1143S 1147S 10.3945/ajcn.2010.28608E 20164318 PMC2844687

[B31] Bain J Testosterone and the aging male: to treat or not to treat? Maturitas 2010 66 16 22 10.1016/j.maturitas.2010.01.009 20153946

[B32] McLaren D Siemens DR Izard J Black A Morales A Clinical practice experience with testosterone treatment in men with testosterone deficiency syndrome BJU Int 2008 102 1142 1146 10.1111/j.1464-410X.2008.07811.x 18540933

[B33] Krause W Mueller U Mazur A Testosterone supplementation in the aging male: which questions have been answered? Aging Male 2005 8 31 38 10.1080/13685530500048872 16106921

[B34] Kaufman JM Vermeulen A The decline of androgen levels in elderly men and its clinical and therapeutic implications Endocr Rev 2005 26 833 876 10.1210/er.2004-0013 15901667

[B35] Kenny AM Prestwood KM Gruman CA Fabregas G Biskup B Mansoor G Effects of transdermal testosterone on lipids and vascular reactivity in older men with low bioavailable testosterone levels J Gerontol A Biol Sci Med Sci 2002 57 M460 M465 10.1093/gerona/57.7.M460 12084809

[B36] Crawford BA Liu PY Kean MT Bleasel JF Handelsman DJ Randomized placebo-controlled trial of androgen effects on muscle and bone in men requiring long-term systemic glucocorticoid treatment J Clin Endocrinol Metab 2003 88 3167 3176 10.1210/jc.2002-021827 12843161

[B37] Kenny AM Prestwood KM Gruman CA Marcello KM Raisz LG Effects of transdermal testosterone on bone and muscle in older men with low bioavailable testosterone levels J Gerontol A Biol Sci Med Sci 2001 56 M266 M272 10.1093/gerona/56.5.M266 11320105

[B38] Morley JE Perry HM 3rd Kaiser FE Kraenzle D Jensen J Houston K Mattammal M Perry HM Jr Effects of testosterone replacement therapy in old hypogonadal males: a preliminary study J Am Geriatr Soc 1993 41 149 152 8426037 10.1111/j.1532-5415.1993.tb02049.x

[B39] Marin P Holmang S Gustafsson C Jonsson L Kvist H Elander A Eldh J Sjostrom L Holm G Bjorntorp P Androgen treatment of abdominally obese men Obes Res 1993 1 245 251 16350577 10.1002/j.1550-8528.1993.tb00618.x

[B40] Sullivan DH Roberson PK Johnson LE Bishara O Evans WJ Smith ES Price JA Effects of muscle strength training and testosterone in frail elderly males Med Sci Sports Exerc 2005 37 1664 1672 10.1249/01.mss.0000181840.54860.8b 16260965

[B41] Svartberg J Agledahl I Figenschau Y Sildnes T Waterloo K Jorde R Testosterone treatment in elderly men with subnormal testosterone levels improves body composition and BMD in the hip Int J Impot Res 2008 20 378 387 10.1038/ijir.2008.19 18480825

[B42] Steidle C Schwartz S Jacoby K Sebree T Smith T Bachand R AA2500 testosterone gel normalizes androgen levels in aging males with improvements in body composition and sexual function J Clin Endocrinol Metab 2003 88 2673 2681 10.1210/jc.2002-021058 12788872

[B43] Kenny AM Kleppinger A Annis K Rathier M Browner B Judge JO McGee D Effects of transdermal testosterone on bone and muscle in older men with low bioavailable testosterone levels, low bone mass, and physical frailty J Am Geriatr Soc 2010 58 1134 1143 10.1111/j.1532-5415.2010.02865.x 20722847 PMC3014265

[B44] Kaufman JM Miller MG Garwin JL Fitzpatrick S McWhirter C Brennan JJ Efficacy and safety study of 1.62% testosterone gel for the treatment of hypogonadal men J Sex Med 2011 8 2079 2089 10.1111/j.1743-6109.2011.02265.x 21492400

[B45] Frederiksen L Hojlund K Hougaard DM Brixen K Andersen M Testosterone therapy increased muscle mass and lipid oxidation in aging men Age (Dordr) 2012 34 145 156 10.1007/s11357-011-9213-9 21347608 PMC3260358

[B46] Legros JJ Meuleman EJ Elbers JM Geurts TB Kaspers MJ Bouloux PM Oral testosterone replacement in symptomatic late-onset hypogonadism: effects on rating scales and general safety in a randomized, placebo-controlled study Eur J Endocrinol 2009 160 821 831 10.1530/EJE-08-0634 19211706

[B47] Pugh PJ Jones RD West JN Jones TH Channer KS Testosterone treatment for men with chronic heart failure Heart 2004 90 446 447 10.1136/hrt.2003.014639 15020527 PMC1768161

[B48] Chapman IM Visvanathan R Hammond AJ Morley JE Field JB Tai K Belobrajdic DP Chen RY Horowitz M Effect of testosterone and a nutritional supplement, alone and in combination, on hospital admissions in undernourished older men and women Am J Clin Nutr 2009 89 880 889 10.3945/ajcn.2008.26538 19144729

[B49] Testosterone treatment of men with alcoholic cirrhosis: a double-blind study The Copenhagen Study Group for Liver Diseases Hepatology 1986 6 807 813 10.1002/hep.1840060502 2875927

[B50] Basaria S Coviello AD Travison TG Storer TW Farwell WR Jette AM Eder R Tennstedt S Ulloor J Zhang A Choong K Lakshman KM Mazer NA Miciek R Krasnoff J Elmi A Knapp PE Brooks B Appleman E Aggarwal S Bhasin G Hede-Brierley L Bhatia A Collins L LeBrasseur N Fiore LD Bhasin S Adverse events associated with testosterone administration N Engl J Med 2010 363 109 122 10.1056/NEJMoa1000485 20592293 PMC3440621

[B51] Hall GM Larbre JP Spector TD Perry LA Da Silva JA A randomized trial of testosterone therapy in males with rheumatoid arthritis Br J Rheumatol 1996 35 568 573 10.1093/rheumatology/35.6.568 8670579

[B52] Sih R Morley JE Kaiser FE Perry HM 3rd Patrick P Ross C Testosterone replacement in older hypogonadal men: a 12-month randomized controlled trial J Clin Endocrinol Metab 1997 82 1661 1667 10.1210/jc.82.6.1661 9177359

[B53] English KM Steeds RP Jones TH Diver MJ Channer KS Low-dose transdermal testosterone therapy improves angina threshold in men with chronic stable angina: a randomized, double-blind, placebo-controlled study Circulation 2000 102 1906 1911 10.1161/01.CIR.102.16.1906 11034937

[B54] Amory JK Watts NB Easley KA Sutton PR Anawalt BD Matsumoto AM Bremner WJ Tenover JL Exogenous testosterone or testosterone with finasteride increases bone mineral density in older men with low serum testosterone J Clin Endocrinol Metab 2004 89 503 510 10.1210/jc.2003-031110 14764753

[B55] Kenny AM Fabregas G Song C Biskup B Bellantonio S Effects of testosterone on behavior, depression, and cognitive function in older men with mild cognitive loss J Gerontol A Biol Sci Med Sci 2004 59 75 78 10.1093/gerona/59.1.M75 14718489

[B56] Svartberg J Aasebo U Hjalmarsen A Sundsfjord J Jorde R Testosterone treatment improves body composition and sexual function in men with COPD, in a 6-month randomized controlled trial Respir Med 2004 98 906 913 10.1016/j.rmed.2004.02.015 15338805

[B57] Brockenbrough AT Dittrich MO Page ST Smith T Stivelman JC Bremner WJ Transdermal androgen therapy to augment EPO in the treatment of anemia of chronic renal disease Am J Kidney Dis 2006 47 251 262 10.1053/j.ajkd.2005.10.022 16431254

[B58] Malkin CJ Pugh PJ West JN van Beek EJ Jones TH Channer KS Testosterone therapy in men with moderate severity heart failure: a double-blind randomized placebo controlled trial Eur Heart J 2006 27 57 64 16093267 10.1093/eurheartj/ehi443

[B59] Merza Z Blumsohn A Mah PM Meads DM McKenna SP Wylie K Eastell R Wu F Ross RJ Double-blind placebo-controlled study of testosterone patch therapy on bone turnover in men with borderline hypogonadism Int J Androl 2006 29 381 391 10.1111/j.1365-2605.2005.00612.x 16390499

[B60] Nair KS Rizza RA O’Brien P Dhatariya K Short KR Nehra A Vittone JL Klee GG Basu A Basu R Cobelli C Toffolo G Dalla MC Tindall DJ Melton LJ 3rd Smith GE Khosla S Jensen MD DHEA in elderly women and DHEA or testosterone in elderly men N Engl J Med 2006 355 1647 1659 10.1056/NEJMoa054629 17050889

[B61] Emmelot-Vonk MH Verhaar HJ Nakhai Pour HR Aleman A Lock TM Bosch JL Grobbee DE van der Schouw YT Effect of testosterone supplementation on functional mobility, cognition, and other parameters in older men: a randomized controlled trial JAMA 2008 299 39 52 10.1001/jama.2007.51 18167405

[B62] Caminiti G Volterrani M Iellamo F Marazzi G Massaro R Miceli M Mammi C Piepoli M Fini M Rosano GM Effect of long-acting testosterone treatment on functional exercise capacity, skeletal muscle performance, insulin resistance, and baroreflex sensitivity in elderly patients with chronic heart failure a double-blind, placebo-controlled, randomized study J Am Coll Cardiol 2009 54 919 927 10.1016/j.jacc.2009.04.078 19712802

[B63] Aversa A Bruzziches R Francomano D Rosano G Isidori AM Lenzi A Spera G Effects of testosterone undecanoate on cardiovascular risk factors and atherosclerosis in middle-aged men with late-onset hypogonadism and metabolic syndrome: results from a 24-month, randomized, double-blind, placebo-controlled study J Sex Med 2010 7 3495 3503 10.1111/j.1743-6109.2010.01931.x 20646185

[B64] Srinivas-Shankar U Roberts SA Connolly MJ O’Connell MD Adams JE Oldham JA Wu FC Effects of testosterone on muscle strength, physical function, body composition, and quality of life in intermediate-frail and frail elderly men: a randomized, double-blind, placebo-controlled study J Clin Endocrinol Metab 2010 95 639 650 10.1210/jc.2009-1251 20061435

[B65] Jones TH Arver S Behre HM Buvat J Meuleman E Moncada I Morales AM Volterrani M Yellowlees A Howell JD Channer KS Testosterone replacement in hypogonadal men with type 2 diabetes and/or metabolic syndrome (the TIMES2 study) Diabetes Care 2011 34 828 837 10.2337/dc10-1233 21386088 PMC3064036

[B66] Ho CC Tong SF Low WY Ng CJ Khoo EM Lee VK Zainuddin ZM Tan HM A randomized, double-blind, placebo-controlled trial on the effect of long-acting testosterone treatment as assessed by the Aging Male Symptoms scale BJU Int 2012 110 260 265 10.1111/j.1464-410X.2011.10755.x 22093057

[B67] Kalinchenko SY Tishova YA Mskhalaya GJ Gooren LJ Giltay EJ Saad F Effects of testosterone supplementation on markers of the metabolic syndrome and inflammation in hypogonadal men with the metabolic syndrome: the double-blinded placebo-controlled Moscow study Clin Endocrinol (Oxf) 2010 73 602 612 10.1111/j.1365-2265.2010.03845.x 20718771

[B68] Hoyos CM Yee BJ Phillips CL Machan EA Grunstein RR Liu PY Body compositional and cardiometabolic effects of testosterone therapy in obese men with severe obstructive sleep apnoea: a randomised placebo-controlled trial Eur J Endocrinol 2012 167 531 541 10.1530/EJE-12-0525 22848006

[B69] Haring R Teumer A Volker U Dorr M Nauck M Biffar R Volzke H Baumeister SE Wallaschofski H Mendelian randomization suggests non-causal associations of testosterone with cardiometabolic risk factors and mortality Andrology 2013 1 17 23 23258625 10.1111/j.2047-2927.2012.00002.x

[B70] Labrie F Cusan L Gomez JL Martel C Berube R Belanger P Belanger A Vandenput L Mellstrom D Ohlsson C Comparable amounts of sex steroids are made outside the gonads in men and women: strong lesson for hormone therapy of prostate and breast cancer J Steroid Biochem Mol Biol 2009 113 52 56 10.1016/j.jsbmb.2008.11.004 19073258

[B71] Labrie F Intracrinology Mol Cell Endocrinol 1991 78 C113 C118 10.1016/0303-7207(91)90116-A 1838082

[B72] Fizazi K Scher HI Molina A Logothetis CJ Chi KN Jones RJ Staffurth JN North S Vogelzang NJ Saad F Mainwaring P Harland S Goodman OB Jr Sternberg CN Li JH Kheoh T Haqq CM de Bono JS Abiraterone acetate for treatment of metastatic castration-resistant prostate cancer: final overall survival analysis of the COU-AA-301 randomised, double-blind, placebo-controlled phase 3 study Lancet Oncol 2012 13 983 992 10.1016/S1470-2045(12)70379-0 22995653

[B73] Scher HI Fizazi K Saad F Taplin ME Sternberg CN Miller K De WR Mulders P Chi KN Shore ND Armstrong AJ Flaig TW Flechon A Mainwaring P Fleming M Hainsworth JD Hirmand M Selby B Seely L de Bono JS Increased survival with enzalutamide in prostate cancer after chemotherapy N Engl J Med 2012 367 1187 1197 10.1056/NEJMoa1207506 22894553

[B74] The Coronary Drug Project Findings leading to discontinuation of the 2.5-mg day estrogen group. The Coronary Drug Project Research Group JAMA 1973 226 652 657 4356847

[B75] Basaria S Davda MN Travison TG Ulloor J Singh R Bhasin S Risk factors associated with cardiovascular events during testosterone administration in older men with mobility limitation J Gerontol A Biol Sci Med Sci 2013 68 153 160 10.1093/gerona/gls138 22562960 PMC3598355

[B76] Idan A Griffiths KA Harwood DT Seibel MJ Turner L Conway AJ Handelsman DJ Long-term effects of dihydrotestosterone treatment on prostate growth in healthy, middle-aged men without prostate disease: a randomized, placebo-controlled trial Ann Intern Med 2010 153 621 632 10.7326/0003-4819-153-10-201011160-00004 21079217

[B77] Kunelius P Lukkarinen O Hannuksela ML Itkonen O Tapanainen JS The effects of transdermal dihydrotestosterone in the aging male: a prospective, randomized, double blind study J Clin Endocrinol Metab 2002 87 1467 1472 10.1210/jc.87.4.1467 11932266

[B78] Ly LP Jimenez M Zhuang TN Celermajer DS Conway AJ Handelsman DJ A double-blind, placebo-controlled, randomized clinical trial of transdermal dihydrotestosterone gel on muscular strength, mobility, and quality of life in older men with partial androgen deficiency J Clin Endocrinol Metab 2001 86 4078 4088 10.1210/jc.86.9.4078 11549629

[B79] Bhasin S Travison TG Storer TW Lakshman K Kaushik M Mazer NA Ngyuen AH Davda MN Jara H Aakil A Anderson S Knapp PE Hanka S Mohammed N Daou P Miciek R Ulloor J Zhang A Brooks B Orwoll K Hede-Brierley L Eder R Elmi A Bhasin G Collins L Singh R Basaria S Effect of testosterone supplementation with and without a dual 5alpha-reductase inhibitor on fat-free mass in men with suppressed testosterone production: a randomized controlled trial JAMA 2012 307 931 939 10.1001/jama.2012.227 22396515 PMC6035750

[B80] Ajayi AA Mathur R Halushka PV Testosterone increases human platelet thromboxane A2 receptor density and aggregation responses Circulation 1995 91 2742 2747 10.1161/01.CIR.91.11.2742 7758179

[B81] Eyben FE Graugaard C Vaeth M All-cause mortality and mortality of myocardial infarction for 989 legally castrated men Eur J Epidemiol 2005 20 863 869 10.1007/s10654-005-2150-0 16283477

[B82] Swerdlow AJ Higgins CD Schoemaker MJ Wright AF Jacobs PA Mortality in patients with Klinefelter syndrome in Britain: a cohort study J Clin Endocrinol Metab 2005 90 6516 6522 10.1210/jc.2005-1077 16204366

[B83] Nguyen PL Je Y Schutz FA Hoffman KE Hu JC Parekh A Beckman JA Choueiri TK Association of androgen deprivation therapy with cardiovascular death in patients with prostate cancer: a meta-analysis of randomized trials JAMA 2011 306 2359 2366 10.1001/jama.2011.1745 22147380

[B84] Bhandari M Busse JW Jackowski D Montori VM Schunemann H Sprague S Mears D Schemitsch EH Heels-Ansdell D Devereaux PJ Association between industry funding and statistically significant pro-industry findings in medical and surgical randomized trials CMAJ 2004 170 477 480 14970094 PMC332713

[B85] Gluud LL Bias in clinical intervention research Am J Epidemiol 2006 163 493 501 10.1093/aje/kwj069 16443796

[B86] Petitti D Commentary: hormone replacement therapy and coronary heart disease: four lessons Int J Epidemiol 2004 33 461 463 10.1093/ije/dyh192 15166191

[B87] Zarrouf FA Artz S Griffith J Sirbu C Kommor M Testosterone and depression: systematic review and meta-analysis J Psychiatr Pract 2009 15 289 305 10.1097/01.pra.0000358315.88931.fc 19625884

[B88] Shamlian NT Cole MG Androgen treatment of depressive symptoms in older men: a systematic review of feasibility and effectiveness Can J Psychiatry 2006 51 295 299 16986819 10.1177/070674370605100505

[B89] Bolona ER Uraga MV Haddad RM Tracz MJ Sideras K Kennedy CC Caples SM Erwin PJ Montori VM Testosterone use in men with sexual dysfunction: a systematic review and meta-analysis of randomized placebo-controlled trials Mayo Clin Proc 2007 82 20 28 17285782 10.4065/82.1.20

[B90] Tracz MJ Sideras K Bolona ER Haddad RM Kennedy CC Uraga MV Caples SM Erwin PJ Montori VM Testosterone use in men and its effects on bone health. A systematic review and meta-analysis of randomized placebo-controlled trials J Clin Endocrinol Metab 2006 91 2011 2016 10.1210/jc.2006-0036 16720668

[B91] Kong A Edmonds P Testosterone therapy in HIV wasting syndrome: systematic review and meta-analysis Lancet Infect Dis 2002 2 692 699 10.1016/S1473-3099(02)00441-3 12409050

[B92] Johns K Beddall MJ Corrin RC Anabolic steroids for the treatment of weight loss in HIV-infected individuals Cochrane Database Syst Rev 2005 4 CD005483 16235407 10.1002/14651858.CD005483 PMC12174972

[B93] D’Agostino RB Sr Vasan RS Pencina MJ Wolf PA Cobain M Massaro JM Kannel WB General cardiovascular risk profile for use in primary care: the Framingham Heart Study Circulation 2008 117 743 753 10.1161/CIRCULATIONAHA.107.699579 18212285

[B94] Menke A Guallar E Rohrmann S Nelson WG Rifai N Kanarek N Feinleib M Michos ED Dobs A Platz EA Sex steroid hormone concentrations and risk of death in US men Am J Epidemiol 2010 171 583 592 10.1093/aje/kwp415 20083549 PMC6596446

[B95] Deloukas P Kanoni S Willenborg C Farrall M Assimes TL Thompson JR Ingelsson E Saleheen D Erdmann J Goldstein BA Stirrups K Konig IR Cazier JB Johansson A Hall AS Lee JY Willer CJ Chambers JC Esko T Folkersen L Goel A Grundberg E Havulinna AS Ho WK Hopewell JC Eriksson N Kleber ME Kristiansson K Lundmark P Lyytikainen LP Large-scale association analysis identifies new risk loci for coronary artery disease Nat Genet 2012 45 25 33 10.1038/ng.2480 23202125 PMC3679547

[B96] Wieland LS Robinson KA Dickersin K Understanding why evidence from randomised clinical trials may not be retrieved from Medline: comparison of indexed and non-indexed records BMJ 2012 344 d7501 10.1136/bmj.d7501 22214757

[B97] Ferreira ML Herbert RD Crowther MJ Verhagen A Sutton AJ When is a further clinical trial justified? BMJ 2012 345 e5913 10.1136/bmj.d7501 22977141

[B98] Pereira TV Ioannidis JP Statistically significant meta-analyses of clinical trials have modest credibility and inflated effects J Clin Epidemiol 2011 64 1060 1069 10.1016/j.jclinepi.2010.12.012 21454050

[B99] LeLorier J Gregoire G Benhaddad A Lapierre J Derderian F Discrepancies between meta-analyses and subsequent large randomized, controlled trials N Engl J Med 1997 337 536 542 10.1056/NEJM199708213370806 9262498

[B100] Kenny AM Bellantonio S Gruman CA Acosta RD Prestwood KM Effects of transdermal testosterone on cognitive function and health perception in older men with low bioavailable testosterone levels J Gerontol A Biol Sci Med Sci 2002 57 M321 M325 10.1093/gerona/57.5.M321 11983727

[B101] Marin P Oden B Bjorntorp P Assimilation and mobilization of triglycerides in subcutaneous abdominal and femoral adipose tissue in vivo in men: effects of androgens J Clin Endocrinol Metab 1995 80 239 243 10.1210/jc.80.1.239 7829619

