# Supplementary material for: Testosterone therapy and cardiovascular events among men: a systematic review and meta-analysis of placebo-controlled randomized trials
Source: BMC Med. 2013 Apr 18;11:108. doi: 10.1186/1741-7015-11-108 (PMC3648456; doi:10.1186/1741-7015-11-108)
Supplement: Additional file 2 — Trials where authors contacted for additional information and responses.[13,35-37,40,42-48,100]. [file 1741-7015-11-108-S2.docx]

Additional file 2: Trials where authors contacted for additional information and responses.

| Author and publication year | Reason for contact | Date | |  |  |
| --- | --- | --- | --- | --- | --- |
|  |  | Contacted | Responded | Response | Action |
| Kenny 2002 [35, 37, 100] | We could not find any reports on this trial giving cardiovascular events in the “Safety” section or the reasons for discontinuation or withdrawal. This trial appears to be shown in a previous meta-analysis with testosterone therapy protecting against cardiovascular related events.[13] Given the trial had 34 in the T group and 33 in P group, this is equivalent to 0 cardiovascular events in the T group and 6 in the P group. | 30 January 2012  10 Feb 2012 | No |  | Study excluded because events inferred from another meta-analysis not confirmed by author |
| Steidle 2003 [42] | Two cardiovascular events are described in the T group (hypertension and coronary artery disease) in a section on events in the treatment group. There is no similar information about the placebo group, so it is unclear whether there were also cardiovascular-related events in the placebo group. | 13 January 2012  10 Feb 2012 | No |  | Study excluded because unclear if events from placebo arm reported |
| Crawford 2003 [36] | The text describes 15 serious adverse events (SAEs) in a study of 51 men comparing nandrolone (17) to testosterone (18) to placebo (16). Of these SAEs, 11 were cardiovascular-related, i.e., myocardial ischemia (4), pulmonary embolism (2), rupture aortic aneurysm (2), cartiod artery thrombosis (1) and cardiomyopathy (2). The text says there was significant different among treatment groups in the distribution of SAEs. A previous meta-analysis shows this study as having what looks like 6 or 7 cardiovascular events in the T group and 0 in the P group.[13] | 12 December 2011  02 February 2012 | Yes | The author replied that because the data was quite old, and in Australia people only have to keep the original data for 7 years, so he could not confirm the number of CVD events. (07 Feb 2012) | Study excluded because events inferred from another meta-analysis not confirmed by author |
| Sullivan 2005 [40] | “Exacerbation of his chronic obstructive pulmonary disease and a non-Q wave myocardial infarction 3 d after a high-intensity workout” Unclear as to study arm where this occurred. | 30 January 2012  10 Feb 2012 | No |  | Study excluded because unclear in which group event occurred. |
| Legros 2009 [46] | The text describes 254 adverse events, but does not give a breakdown of adverse events or serious adverse events by study arm. The paper only gives deaths by study arm. | 12 December 2011  10 February 2012 | Yes | Transferred request at 15 Dec 2011 but no response afterward | Study included with deaths only |
| Chapman 2009 [48] | Deaths are not given by cause and study arm, only hospitalisations | 30 January 2012 | Yes | Author additionally reported one death from myocardial infarction in the testosterone arm. | Study included |
| Kenny 2010 [43] | The study flow chart gives 2 cardiac withdrawals in the testosterone group (CHF and leaky value) and 4 cardiac withdrawals in the placebo group (open heart surgery, myocardial infarction, aneurysm and CHF). The ‘Safety evaluation” section also gives 3 deaths from stroke without giving study arm. The same section also says that “Seven individuals withdrew from treatment for signs and symptoms consistent with cardiac disease, including myocardial infarction (n=2), congestive heart failure (n=3), and significant lower extremity edema (n=2).” | 18 January 2012  10 Feb 2012 | No |  | Study excluded because impossible to reconcile information from different parts of the paper. |
| Pugh 2004 [47] | The text describes “One patient in the active group was admitted to hospital with breathlessness after eight weeks of treatment”, which seems like a cardiovascular disease but could not confirm. | 12 December 2011  02 February 2012 | Yes | Replied at 13 Dec 2011.  Question not answered | Study excluded as diagnosis of event uncertain. |
| Kaufman 2011 [44] | The text says “19 cardiovascular-related events were recorded in 23 patients” without giving study arm, whilst the table of “Incidence of treatment-emergent adverse events in **>**2% of the subjects n (%)” gives 11 events under a heading of “Vascular disorders”. | 01 March 2012  08 March 2012 | No |  | Study included using events recorded as “Vascular disorders” |
| Frederiksen [45] 2012 | The text says “Two SAE occurred in two participants and included a single possible treatment-related event (venous thrombosis in the leg, hematocrit within the normal range) and a single non-related event (car accident).” | 23 January 2013  04 February 2013 | No |  | Study excluded as uncertain whether event occurred in placebo or treatment arm. |

T testosterone, P placebo
