# Supplementary material for: Testosterone therapy and cardiovascular events among men: a systematic review and meta-analysis of placebo-controlled randomized trials
Source: BMC Med. 2013 Apr 18;11:108. doi: 10.1186/1741-7015-11-108 (PMC3648456; doi:10.1186/1741-7015-11-108)
Supplement: Additional file 3 — Quality assessment of the selected placebo-controlled RCTs of the effects of testosterone therapy on cardiovascular-related events (CRE) [19],[26],[39],[41],[44],[46],[48]-[68]. [file 1741-7015-11-108-S3.docx]

Additional file 3: Quality assessment of the selected placebo-controlled RCTs of the effects of testosterone therapy on cardiovascular-related events (CRE).

| Author and publication year | Randomization | Treatment allocation concealed | Group similarity | Eligibility listed | CRE Outcome assessor blinded | Care provider blinded | Subject masked | Point estimates and variability for outcome | ITT of main results | CRE definition pre-specified or assessed before study unblinded | Table showing CRE by study arm |
| --- | --- | --- | --- | --- | --- | --- | --- | --- | --- | --- | --- |
| The Copenhagen Study Group for Liver Diseases [49] 1986 | Yes | Yes | Yes | Yes | Yes | Yes | Yes | Yes | Yes | Yes | Yes |
| Marin [39] 1993 | Yes | Yes | Yes | Yes | NR | NR | Yes | No | No | NR | No |
| Hall [51] 1996 | Yes | NR | No | Yes | NR | NR | Yes | No | No | NR | No |
| Sih [52] 1997 | Yes | Yes | Yes | Yes | NR | NR | Yes | No | No | NR | No |
| English [53] 2000 | Yes | Yes | Yes | Yes | NR | NR | Yes | No | No | NR | No |
| Snyder [19] 2001 | Yes | Yes | No | Yes | NR | Yes | Yes | Yes | No | NR | Yes |
| Amory [54] 2004 | Yes | Yes | Yes | Yes | NR | Yes | Yes | No | Yes | NR | No |
| Kenny [55] 2004 | Yes | NR | No | Yes | NR | NR | Yes | No | No | NR | No |
| Svartberg [56] 2004 | Yes | Yes | Yes | No | NR | NR | Yes | No | No | NR | No |
| Brockenbrough [57] 2006 | Yes | Yes | No | Yes | NR | Yes | Yes | No | Yes | NR | Yes |
| Malkin [58] 2006 | Yes | Yes | Yes | Yes | NR | NR | Yes | No | Yes | NR | Yes |
| Merza [59] 2006 | Yes | NR | Yes | Yes | NR | NR | Yes | No | No | NR | No |
| Nair [60] 2006 | Yes | Yes | Yes | Yes | NR | NR | Yes | No | No | NR | Yes |
| Emmelot-Vonk [61] 2008 | Yes | Yes | Yes | Yes | Yes | Yes | Yes | No | Yes | Yes | Yes |
| Svartberg [41] 2008 | Yes | NR | Yes | Yes | NR | Yes | Yes | No | No | NR | No |
| Caminiti [62] 2009 | Yes | NR | Yes | Yes | NR | NR | Yes | No | No | NR | No |
| Chapman [48] 2009 | yes | NR | yes | yes | NR | NR | yes | No | no | NR | No |
| Legros [46] 2009 | yes | NR | yes | yes | NR | NR | yes | no | no | NR | no |
| Aversa [63] 2010 | Yes | NR | Yes | Yes | NR | NR | Yes | No | No | NR | No |
| Basaria [50] 2010 | Yes | Yes | Yes | Yes | Yes | Yes | Yes | No | No | Yes | Yes |
| Srinivas-Shankar [64] 2010 | Yes | Yes | Yes | Yes | NR | Yes | Yes | No | Yes | NR | No |
| Kalinchenko [67] 2010 | yes | yes | yes | yes | NR | yes | yes | no | no | NR | no |
| Jones [65] 2011 | Yes | Yes | Yes | Yes | Yes | Yes | Yes | No | Yes | NR | No |
| Ho [66] 2011 | Yes | Yes | Yes | Yes | Yes | Yes | Yes | No | No | NR | no |
| Kaufman [44] 2011 | Yes | Yes | Yes | Yes | NR | Yes | Yes | No | No | NR | Yes |
| Hoyos [68] 2012 | Yes | Yes | Yes | Yes | NR | Yes | Yes | No | Yes | NR | Yes |
| Spitzer [26] 2012 | Yes | Yes | Yes | Yes | Yes | Yes | Yes | No | Yes | NR | Yes |

NR=not reported, CRE= cardiovascular-related events
