# Supplementary material for: Testosterone therapy and cardiovascular events among men: a systematic review and meta-analysis of placebo-controlled randomized trials
Source: BMC Med. 2013 Apr 18;11:108. doi: 10.1186/1741-7015-11-108 (PMC3648456; doi:10.1186/1741-7015-11-108)
Supplement: Additional file 4 — Description of cardiovascular-related events in the selected placebo-controlled RCTs [19],[26],[39],[41],[44],[46],[48]-[67],[101]. [file 1741-7015-11-108-S4.docx]

Additional file 4: Description of cardiovascular-related events in the selected placebo-controlled RCTs.

| Author and publication year | Description of events and type of event | | Count | |  | Count of | | |
| --- | --- | --- | --- | --- | --- | --- | --- | --- |
|  |  | Serious | T | P | Comment | Men | Events |  |
| The Copenhagen Study Group for Liver Diseases [49] 1986 | Death from bleeding esophageal varices  Death from acute myocardial infarction  Thrombosis | Y  Y  N | 12  1  3 | 5  0  0 | Unclear whether thrombosis occurred in the participants who died of other cardiovascular causes, so not included as men, but included as events. | 13/5 | 16/5 |  |
| Marin [39] 1993 | Splanchnic venous thrombosis | N | 1 | 0 | Number of subjects by study arm obtained from Marin[101] 1995 | 1/0 |  |  |
| Hall [51]1996 | Hypertension  Cerebrovascular accident | N  Y | 0  0 | 1  1 |  | 0/2 |  |  |
| Sih [52] 1997 | uncontrolled atrial fibrillation with congestive heart failure  Stroke | Y  Y | 1  0 | 0  1 |  | 1/1 | 2/1 |  |
| English [53] 2000 | Myocardial infarction  Early elective coronary angioplasty  Myocardial infarction when awaiting coronary revascularization | Y  Y  Y | 1  1  1 | 0  0  0 | First two events are recorded as withdrawals, 3^rd^ event is given in the ‘Safety’ section, unclear if it is the same event as one of the withdrawals. Counted as the same event. | 2/0 |  |  |
| Synder [19] 2001 | Myocardial infarction  Coronary bypass graft surgery  Arrhythmia  Other vascular events | Y  Y  Y  Y | 2  2  3  2 | 1  2  1  1 | All events verified against hospital records |  | 9/5 |  |
| Amory [54] 2004 | Cerebral hemorrhage | Y | 1 | 0 | An additional man developed sleep apnea in the T group | 1/0 |  |  |
| Kenny [55] 2004 | Cerebral vascular accident | Y | 0 | 1 |  | 0/1 |  |  |
| Svartberg [56] 2004 | Death from probable myocardial infarction | Y | 0 | 1 |  | 0/1 |  |  |
| Brockenbrough [57] 2006 | Cardiac disorders involving death  Cardiac disorders not involving death  Access thrombosis | Y  N  N | 3  4  2 | 1  2  6 | Cardiac disorders included 1 death from stroke in the P group and 3 cardiovascular deaths in the T group. | 9/9 |  |  |
| Malkin [58] 2006 | Arrhythmia  Stroke  Unstable angina pectoris  Hospitalized for exacerbation of heart failure | Y  Y  Y  Y | 0  1  1  2 | 2  0  0  2 | A sudden death also occurred in the P group with unclear cause | 4/4 |  |  |
| Merza [59] 2006 | Angina | N | 0 | 1 |  | 0/1 |  |  |
| Nair [60] 2006 | Ascending aorta dilatation  CAD Stent Placement  Chest pain  Coronary Artery bypass graft  Coronary Artery Disease  Frequent Ventricular Ectopics  Hypotension  Phlebitis  Triple Bypass Surgery | N  Y  N  Y  Y  N  N  N  Y | 1  0  0  1  2  1  0  1  1 | 2  1  1  0  0  0  1  1  0 |  | 5/5 | 7/6 |  |
| Emmelot-Vonk [61] 2008 | Cardiovascular complaints | N | 8 | 3 | Specific types of cardiovascular event not given  Includes one man who withdrew from the T group because of a cardiovascular complaint before the 3 month visit, and so was not included in the analysis presented. |  | 8/3 |  |
| Svartberg [41] 2008 | Death from cardiac arrhythmia | Y | 1 | 0 |  | 1/0 |  |  |
| Caminiti [62] 2009 | Worsening heart failure without hospital stay  Worsening heart failure with hospital stay | N  Y | 1  1 | 1  0 |  | 2/1 |  |  |
| Chapman [48] 2009 | Death from myocardial infarction  Hospitalisation for myocardial infarction | Y  Y | 1  0 | 0  1 | 1 ischemic chest pain in P group not counted | 1/1 | 1/1 |  |
| Legros [46] 2009 | Death from arrhythmia and hypertrophic cardiomyopathy | Y | 1 | 0 | Another man allocated to T died of cardiac arrest 3 months after the end of the study. This event was not included. | 1/0 |  |  |
| Aversa [63] 2010 | Acute myocardial infarction | Y | 0 | 1 | Unclear when the event occurred, i.e., when placebo group were on placebo or after they were switched to T, event included. | 0/1 |  |  |
| Basaria [50] 2010 | Acute coronary syndrome and chest pain  Syncope  Myocardial infarction  Angioplasty and coronary artery bypass  Peripheral edema  Ectopy on ECG  Left ventricular strain pattern during exercise testing  ST-segment depression during exercise testing  Elevated blood pressure and atrial fibrillation  Elevated blood pressure  Chest pain  Atrial fillibration  Stroke  Tachycardia with fatigue  Tachycardia  Death suspected myocardial infarction  Congestive heart failure exacerbation  Arrhythmia –ectopy noted on CG before exercise testing  Carotid bruit and carotid-artery plaque identified on ultra-sonography | Y  N  Y  Y  N  N  N  N  N  N  N  N  Y  N  N  Y  Y  Y  N | 2  2  2  1  5  1  1  1  1  2  1  2  1  1  0  1  1  0  0 | 0  1  0  0  0  0  0  0  0  1  0  0  0  0  1  0  0  1  1 | “Two men, both in the testosterone group, underwent elective vascular procedures during treatment that were not included in these analyses since they were deemed to have been related to preexisting conditions: Subject 22 underwent elective surgery to repair an aortic aneurysm, and another participant, not listed in this table, underwent prescheduled elective angioplasty and placement of a stent in his leg owing to peripheral vascular disease”. These 2 events were not included. | 23/5 | 25/5 |  |
| Srinivas-Shankar [64] 2010 | Acute myocardial infarction  Died of ruptured aneurysm  Angina  Pulmonary embolism (given as a serious adverse event)  Died of constrictive pericarditis  Abdominal aneurysm (surgery)  Heart failure (given as a serious adverse event) | Y  Y  N  Y  Y  Y  Y | 0  0  1  1  1  1  1 | 1  1  0  0  0  0  0 | Events taken from the withdrawals on flowchart and serious adverse events in text. Most adverse events can be found among the withdrawals. One serious adverse event (heart failure) is not listed as withdrawal. One reason for withdrawal (angina) is not listed as a serious adverse event. These were counted as separate events. |  | 5/2 |  |
| Kalinchenko [67] 2010 | angina onset  died, cause myocardial infarction | N  Y | 0  0 | 1  1 |  | 0/2 |  |  |
| Ho [66] 2011 | Died myocardial infarction | Y | 1 | 1 | 1 chest pain in each group not counted | 1/1 |  |  |
| Jones [65] 2011 | Died of myocardial infarction  No details given | Y  N | 0  5 | 1  11 | No actual numbers given, but inferred from the p-value. No information on type of event given, except that one man from the placebo group died of a myocardial infarction |  | 5/12 |  |
| Kaufman [44] 2011 | Myocardial infarction  Other vascular events | Y  N | 1  10 | 0  0 | Of these 11 events 6 described as hypertension, but 19 cardiovascular-related events given elsewhere in the text without giving study arm |  | 11/0 |  |
| Hoyos [68] 2012 | Cardiovascular events | N | 1 | 0 | Cardiac |  | 1/0 |  |
| Spitzer [26] 2012 | Cardiovascular involving hospitalization  Cardiovascular not involving hospitalization | Y  N | 1  3 | 0  2 | Specific types of cardiovascular event not given |  | 4/2 |  |

T testosterone, P placebo
